# The Relation of Clinic and Ambulatory BP with the Risk of Cardiovascular Events and All-Cause Mortality among Patients on Peritoneal Dialysis

**DOI:** 10.3390/jcm10112232

**Published:** 2021-05-21

**Authors:** Panagiotis I. Georgianos, Vasilios Vaios, Pantelis E. Zebekakis, Vassilios Liakopoulos

**Affiliations:** Peritoneal Dialysis Unit, 1st Department of Internal Medicine, AHEPA Hospital, School of Medicine, Aristotle University of Thessaloniki, GR54636 Thessaloniki, Greece; pangeorgi@yahoo.gr (P.I.G.); vvaios_85@yahoo.gr (V.V.); pzempeka@auth.gr (P.E.Z.)

**Keywords:** ambulatory BP, cardiovascular events, clinic BP, mortality, peritoneal dialysis

## Abstract

Large observational studies showed a U-shaped association of clinic blood pressure (BP) with mortality among patients undergoing peritoneal dialysis (PD). Whether ambulatory BP provides a more direct risk signal in this population remains unknown. In a prospective cohort of 108 PD patients, standardized clinic BP was recorded at baseline with the validated device HEM-705 (Omron, Healthcare, Bannockburn, IL, USA) and 24-h ambulatory BP monitoring was performed using the Mobil-O-Graph monitor (IEM, Stolberg, Germany). Over a median follow-up of 16 months (interquartile range: 19 months), 47.2% of the overall population reached the composite outcome of non-fatal myocardial infarction, non-fatal stroke, or all-cause death. In Cox-regression analysis, systolic but not diastolic BP was prognostically informative. Compared with the reference quartile 1 of 24-h systolic BP (SBP), the multivariate-adjusted hazard ratio for the composite outcome was 1.098 (95% confidence interval (CI): 0.434–2.777) in quartile 2, 1.004 (95% CI: 0.382–2.235) in quartile 3 and 2.449 (95% CI: 1.156–5.190) in quartile 4. In contrast, no such association was observed between increasing quartiles of clinic SBP and composite outcome. The present study shows that among PD patients, increasing ambulatory SBP is independently associated with higher risk of adverse cardiovascular events and mortality, providing superior prognostic information than standardized clinic SBP.

## 1. Introduction

Hypertension is an established cardiovascular risk factor both in the general population and in patients with chronic kidney disease not yet on dialysis [1]. Unlike the direct and linear relation of blood pressure (BP) with clinical outcomes in non-dialysis populations, longitudinal studies showed an inverse association between clinic BP and mortality among end-stage-renal-disease (ESRD) patients undergoing long-term peritoneal dialysis (PD) [2,3]. Similarly, large epidemiological studies showed consistently a U-shaped or J-shaped association of predialysis and postdialysis BP with the risk of all-cause death in patients receiving maintenance hemodialysis [4,5]. This phenomenon of lower BP to be paradoxically associated with excess mortality risk has been described as “reverse epidemiology” of hypertension, raising concerns and uncertainty on whether hypertension in the ESRD population is an independent risk factor that should be aggressively controlled [6,7].

The reverse epidemiology of hypertension among patients on dialysis requires a closer examination. Prior studies revealed that confounding factors with an opposing effect on BP, such as underlying congestive heart failure (CHF), inflammation, or the level of illness, magnify the inverse relation of BP with mortality, limiting the ability of hypertension to predict risk [8]. Other studies showed that among patients on hemodialysis, elevated BP recorded over the interdialytic interval was directly associated with excess risk of all-cause death [9,10]. In sharp contrast, neither routine nor standardized BP recordings taken shortly before or after dialysis were prognostically informative [9,10]. Thus, the timing or the technique of BP measurement appears to be another factor that modifies the risk-association of BP with mortality.

Based on the above observations, we hypothesized that among patients on PD, ambulatory BP provides a more precise reflection of the patient’s actual BP load over the entire 24-h period and may therefore be of greater prognostic significance than clinic BP. Accordingly, the aim of this study was to explore for first time the association of BP with the risk of future cardiovascular events and all-cause mortality using standardized clinic BP recordings and the reference-standard method of ambulatory BP monitoring (ABPM) as risk predictors in a prospective cohort of 108 prevalent PD patients.

## 2. Materials and Methods

### 2.1. Study Population

The cross-sectional data on part of this cohort have previously been reported elsewhere [11]. Adult ESRD patients receiving PD for at least 3 months in 3 dialysis centers of Northern Greece with a valid 24-h ABPM evaluation at baseline were enrolled in this prospective observational study. Patients were not eligible in the study for the following reasons: (i) chronic atrial fibrillation or other chronic cardiac arrhythmia; (ii) recent episode of acute peritonitis or other infectious/bleeding complications over the previous month; (iii) hospitalization for acute myocardial infarction (MI), unstable angina or acute stroke within the last month before enrolment; (iv) body mass index >40 kg/m^2^; (v) arteriovenous fistula in both arms that was formerly used as vascular access for hemodialysis; (vi) patients who had a change either in the PD regimen or in the prescribed antihypertensive medications within 2 weeks before enrolment were also excluded from the study.

The protocol procedures of our study were accordant with the Declaration of Helsinki and its latest Amendments and all patients gave written informed consent before enrolment. The protocol of our study received approval by the ethics committee of School of Medicine, Aristotle University of Thessaloniki (code of approval: 448/18-07-18).

### 2.2. Predictors

#### 2.2.1. Clinic BP

Clinic BP was recorded at baseline visit by nurses trained in this technique under standardized conditions with the validated self-inflating oscillometric device HEM-705 CP (Omron, HealthCare, Bannockburn, IL, USA) [12]. In detail, 3 automatic BP recordings were obtained in the non-dominant arm with 1-min interval between them after a 5-min seated rest in a quiet room, according to the 2018 guidelines of the European Society of Hypertension/European Society of Cardiology (ESH/ESC) [1]. The average of these 3 BP recordings was calculated and was used as risk predictor in statistical analysis.

#### 2.2.2. 24-H Ambulatory BP

After the completion of baseline assessment at clinic, all patients underwent ABPM with the oscillometric Mobil-O-Graph device (I.E.M. GmbH, Stolberg, Germany). A brachial cuff of appropriate size was fitted to the non-dominant arm and ABPM was performed for 24 h. The BP-detection unit of this device was validated according to the criteria of ESH/ESC and British Society of Hypertension [13,14]. Comparative studies showed that brachial BP recordings obtained with the Mobil-O-Graph monitor under static or ambulatory conditions exhibit acceptable agreement with BP measurements taken with other commercially available and validated ABPM devices [15]. Ambulatory BP was recorded every 20 min during day (07:00–22:59) and every 30 min during night (23:00–06:59). ABPM was considered accurate if >80% of BP readings were valid with ≤2 nonconsecutive day-hours with <2 valid readings and ≤1 night-hour without valid reading [16]. Patients with incomplete or invalid recordings were asked to repeat ABPM within the next week. The present study included only patients with adequate ABPM data at baseline.

### 2.3. Outcome

The primary outcome of our study was prespecified as the composite of time to first occurrence of non-fatal MI, non-fatal stroke, or death from any cause. Patients were prospectively followed up from the day that they successfully completed the baseline evaluations through 28 February 2021. Patients were censored on the date that they received their last PD treatment, if they received a kidney transplant or when they were switched to hemodialysis. The adjudication of deaths and non-mortal cardiovascular events was performed by an independent member of the investigator team (V.V.) after review of medical records that were provided by the 3 participating PD centers.

### 2.4. Statistical Analysis

Continuous variables are presented as mean ± standard deviation (mean ± SD) or median (range), according to the normality of the distribution of each variable assessed using the Kolmogorov–Smirnov test. Categorical data are expressed as absolute frequencies and percentages. The study population was divided into quartiles according to the level of clinic and 24-h ambulatory systolic BP (SBP). Differences in baseline characteristics among quartiles were evaluated using one-way analysis of variance (ANOVA) for continuous variables or with the chi-squared (χ2) test for categorical variables, respectively. Kaplan–Meier curves were created, and the log-rank test was applied to explore the equality in the risk of the primary composite outcome between quartiles of clinic and 24-h ambulatory SBP. Cox proportional hazards regression analysis was applied to explore the prognostic association of baseline BP with the prespecified composite outcome of non-fatal MI, non-fatal stroke, or all-cause death. The analysis was initially performed using unadjusted models. Subsequently, we generated multivariate models that provided adjustment for the following variables: age, sex, time on PD, mode of PD (continuous ambulatory vs. automated), diabetic status, history of pre-existing cardiovascular disease (defined as previous history of MI, coronary artery bypass grafting/angioplasty, or prior stroke), BP medication use (yes vs. no), the presence of substantial residual diuresis (yes vs. no), hemoglobin, and serum albumin levels at baseline. To calculate the adjusted hazard ratios (HRs), continuous variables (age, dialysis vintage, hemoglobin and serum albumin) were centered at their group means. All analyses were performed with the Statistical Package for Social Sciences version 23.0 (SPSS Inc., Chicago, IL, USA). The probability values reported are 2-sited and considered to be statistically significant at *p* < 0.05.

## 3. Results

**Figure 1:**
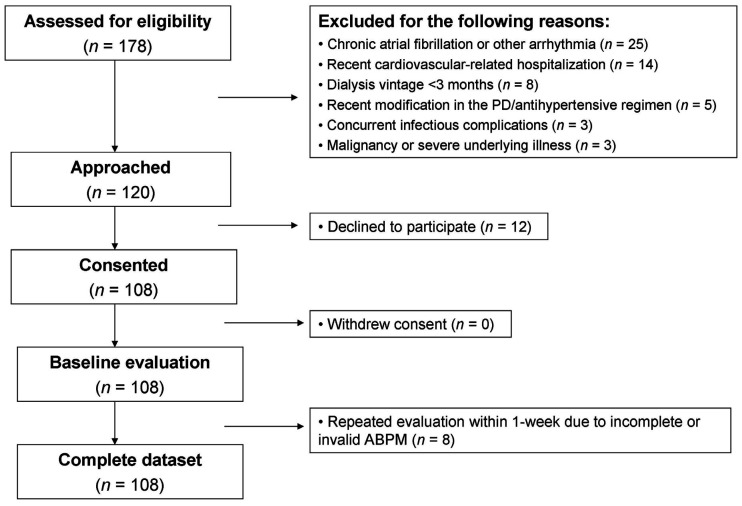
Study flow diagram of patient enrolment.

As shown in Figure 1, between July 2017 and September 2020, 178 prevalent PD patients from 3 dialysis centers of Northern Greece were screened for eligibility in this prospective cohort study. Of these, 58 patients were excluded because they did not fulfill the prespecified inclusion/exclusion criteria. Of the 120 patients approached, 108 provided informed written consent and successfully completed the baseline evaluation with standardized clinic and 24-h ambulatory BP recordings. The prespecified composite outcome of non-fatal MI, non-fatal stroke, or all-cause death occurred in 51 patients (47.2% of the overall study population) over a median follow-up of 16 months (interquartile range: 19 months).

The baseline demographic and clinical characteristics of study participants are shown in Table 1. The overall study population consisted of 70 male and 38 female PD patients who had an average age of 62.8 ± 15.8 years and a mean dialysis vintage of 25.9 ± 28.5 months. The mean standardized clinic BP was 132.9/78.0 mmHg and the mean 24-h ambulatory BP was 126.7/77.6 mmHg. Most of the patients (90.7%) were being treated with at least 1 antihypertensive medication at study enrolment. Patients within the highest quartile of 24-h ambulatory SBP were receiving more commonly treatment with a renin-angiotensin-system blocker or a calcium-channel-blocker, whereas β-blocker use did not significantly differ among quartiles. Overall, 44.4% of the patients had a previous history of MI, coronary artery bypass grafting/angioplasty, or stroke. Not surprisingly, the distribution of pre-existing cardiovascular disease differed significantly among quartiles and cardiovascular comorbidities were more common among patients who were stratified in the lowest quartile of 24-h ambulatory SBP. When the patients were stratified into quartiles according to the levels of standardized clinic SBP at baseline (Table S1), there was a weak agreement between clinic and 24-h ambulatory BP recordings in the classification of the severity of hypertension (k-statistic: 0.395, *p* < 0.001).

**Figure 2:**
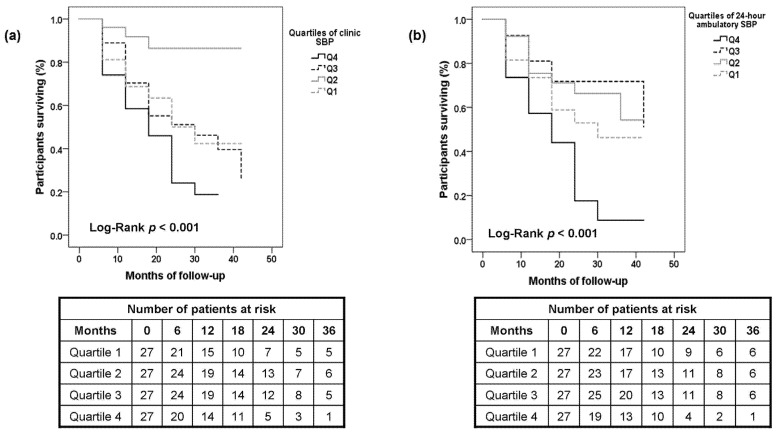
Kaplan–Meier survival curves for the association of increasing quartiles of (**a**) standardized clinic SBP and (**b**) 24-h ambulatory SBP with the composite outcome of non-fatal MI, non-fatal stroke, and all-cause death.

Figure 2 shows the Kaplan–Meier survival curves depicting the relation of baseline SBP measured using standardized clinic and 24-h ambulatory BP recordings with the prespecified composite outcome. The diastolic component of BP was consistently of no prognostic significance regardless of the method of BP measurement (data not shown). In contrast, in Kaplan–Meier survival analysis, the log-rank test demonstrated a significant difference in the risk of the composite outcome between quartiles of clinic and 24-h ambulatory SBP (*p* < 0.001). 

Similarly, in univariate Cox-regression analysis, a significant association was observed between quartiles of clinic SBP and composite outcome (model χ2: 16.5, *p* = 0.001) as well as between quartiles of 24-h ambulatory SBP and composite outcome (model χ2: 18.4, *p* < 0.001) (Table 2). However, the pattern of risk-association was dependent on the technique of BP measurement. Compared with the reference quartile 1 of clinic SBP, patients stratified in quartile 2 had significantly lower risk of the composite outcome (HR: 0.201; 95% confidence interval (CI): 0.057–0.711), whereas the HR for the composite outcome was not significantly higher either in quartile 3 (HR: 1.028; 95% CI: 0.486–2.156) or in quartile 4 (HR: 1.750; 95% CI: 0.851–3.598). Conversely, compared with reference quartile 1 of 24-h ambulatory SBP, the risk of the composite outcome was not significantly different in quartile 2 (HR: 0.667; 95% CI: 0.280–1.586) and in quartile 3 (HR: 0.558; 95% CI: 0.228–1.367), bit it was 2.24-fold higher in quartile 4 (HR: 2.240; 95% CI: 1.103–4.547). When the analysis was adjusted for age, sex, dialysis vintage, history of pre-existing cardiovascular disease, the presence of residual diuresis and other risk factors, the inverse association of clinic SBP with the composite outcome was not substantially modified. Similarly, multivariate adjustment for the same risk factors did not mitigate the association of increasing 24-h ambulatory SBP with greater risk of the composite outcome (Table 2).

Table 3 shows a significant association of increasing quartiles of daytime and nighttime ambulatory SBP with higher risk of future cardiovascular events and all-cause death. Compared with the reference quartile 1 of daytime SBP, the multivariate-adjusted HR for the composite outcome was 0.854 (95% CI: 0.313–2.328) in quartile 2, 1.061 (95% CI: 0.427–2.637) in quartile 3 and 2.631 (95% CI: 1.247–5.535) in quartile 4. A more direct dose-response relationship was observed when nighttime SBP was used as risk predictor. Compared with the reference quartile 1, the multivariate-adjusted HR for the composite outcome was 0.594, 1.555 and 2.305 in quartiles 2, 3, and 4 of nighttime SBP, respectively.

## 4. Discussion

Large observational studies using conventional clinic BP recordings as risk predictors showed a “reverse epidemiology” of hypertension among patients undergoing long-term PD [2,3,7,17]. The present study incorporated for first time standardized clinic BP recordings in conjunction with the reference-standard method of ABPM aiming to explore more objectively the relation between BP and risk of adverse cardiovascular events or all-cause death in the PD population. The main findings of our study are as follows: (i) in accordance with the U-shaped or J-shaped risk-association of clinic BP with mortality seen in prior observational studies [3,4,5,18], the HR for the composite outcome was not significantly higher among patients stratified in the highest quartile of clinic SBP as compared with the reference quartile 1; (ii) whereas increasing clinic SBP did not provide a direct risk signal, patients stratified in the highest quartile of 24-h ambulatory SBP had 2.45-fold higher risk of the composite outcome as compared with the reference quartile 1; (iii) a dose-response relationship was also observed between increasing quartiles of both daytime and nighttime ambulatory SBP and the risk of the composite outcome; (iv) these risk-associations persisted in multivariate Cox-regression analysis that provided adjustment for age, sex, dialysis vintage, pre-existing cardiovascular disease, the presence of residual diuresis at baseline and other established risk factors.

These observations are in line with the results of 2 separate cohort studies showing that the timing or the technique of BP measurement inserts variation in the prognostic association of BP with mortality in the ESRD population [9,10]. In the first study, 150 hemodialysis patients underwent a baseline evaluation with 4 different methods of BP measurement [10]. Over a median follow-up of 24 months, each 1-SD higher home SBP was associated with 35% higher risk of all-cause death (HR: 1.35; 95% CI: 0.99–1.84). Similarly, each 1-SD higher interdialytic ambulatory SBP was associated with 46% higher risk of all-cause death (HR: 1.46; 95% CI: 1.09–1.94) [10]. In contrast, neither routine nor standardized dialysis-unit BP was predictor of mortality. In a much larger study, 326 hemodialysis patients underwent a similar baseline assessment of BP and were prospectively followed up over a median period of 32 months [9]. In multivariate Cox-regression analysis, compared with the reference quartile 1 of interdialytic ambulatory SBP, the HR for all-cause death was 2.51 (95% CI: 1.27–4.95) in quartile 2, 3.43 (95% CI: 1.73–6.79) in quartile 3 and 2.62 (95% CI: 1.33–5.17) in quartile 4 [9]. Conversely, no such dose-response relationship was observed when either routine or standardized pre- and postdialysis BP recordings were used as risk predictors [9].

The diverse prognostic association of clinic versus ambulatory SBP that was evident in the present study could be theoretically attributed to the greater number of BP recordings that the technique of ABPM provides. However, we believe that this potential explanation is an oversimplification of the overall value of ABPM in the assessment of hypertension. This issue was illustrated in a prior observational study showing that among hemodialysis patients, interdialytic ambulatory SBP retained its strong prognostic association with all-cause mortality, even when a small subset of randomly selected ambulatory BP measurements was averaged and used as risk predictors [19]. Therefore, it appears that it is the location—not simply the quantity of BP measurements—the factor that modifies the risk-association of BP with mortality [8].

The superior predictive value of ambulatory over clinic BP may be explained by the fact that ABPM provides a more precise reflection of the patient’s actual BP burden over the entire 24-h period [6,16,20]. Even when clinic BP is recorded under standardized conditions, as done in the present study, it is still subjected to bias arising from the white-coat effect (i.e., high BP only in the clinic); in contrast, the white-coat effect is fully eliminated with the use of ABPM [6,16,20]. Furthermore, the technique of ABPM facilitates the identification of masked hypertension (i.e., normal clinic BP but high BP outside of the clinic) [21]. Other advantages of ABPM also exist. Although clinic BP recordings are typically obtained while the patient is sitting and resting, ABPM provides the opportunity to record BP during periods of activity [6,16,20]. With the use of ABPM, BP can be recorded during periods of sleep, enabling the detection of nocturnal hypertension and non-dipping BP patterns [6,16,20]. These BP phenotypes are very common among patients on dialysis and have been associated with increased risk of cardiovascular morbidity and mortality [22,23,24]. Similarly, increasing nighttime SBP was directly associated with excess risk of the primary composite outcome in the present study.

Strength of our study is the careful assessment of BP at baseline with the use of standardized clinical BP recordings and the reference-standard method of ABPM. However, the present work has also some limitations that need to be acknowledged. First, the observational design of this study precludes the opportunity to derive direct cause-and-effect associations between BP and risk of future cardiovascular events and all-cause death. However, this limitation is commonly shared across all prior observational studies that attempted to explore the prognostic significance of BP in patients on dialysis. Second, patients with infectious/bleeding complications shortly before study enrolment or those with a recent hospitalization due to acute coronary syndrome or acute stroke were excluded from our analysis. Therefore, larger studies also including patients with more severe underlying illness are warranted to confirm whether our observations are applicable to the whole spectrum of the PD population. Third, even though the survival analysis was adjusted for several established risk factors, we acknowledge that the possibility of residual confounding is still not fully eliminated. Finally, since the assessment of clinic and ambulatory BP was performed only in a single occasion at baseline, our analysis could not provide time-varying risk-associations between BP and clinical outcomes.

## 5. Conclusions

In conclusion, the present study shows that among patients on long-term PD, increasing ambulatory SBP was associated with increased risk of adverse cardiovascular events and all-cause death independently from several established risk factors. In contrast, the HR for the composite outcome was not significantly higher in the highest quartile of clinic SBP as compared with the reference quartile 1, suggesting that increasing clinic SBP could not provide a direct risk signal. Future studies are warranted to fully elucidate whether the adequate assessment of hypertension with the use of ABPM can improve cardiovascular risk stratification in the PD population.

## Figures and Tables

**Table 1:** Baseline characteristics of study participants.

Parameter	Overall	Quartile 1	Quartile 2	Quartile 3	Quartile 4	*p* Value
Range of 24-h ambulatory SBP (mmHg)	-	<114.0	114–126	126–140.8	>140.8	-
*N*	108	27	27	27	27	-
24-h ambulatory SBP (mmHg)	126.7 ± 18.4	104.6 ± 7.6	119.5 ± 3.9	132.2 ± 4.3	150.6 ± 10.4	<0.001
24-h ambulatory DBP (mmHg)	77.6 ± 12.1	66.3 ± 7.8	74.1 ± 8.1	82.9 ± 8.8	87.1 ± 11.3	<0.001
Age (years)	62.8 ± 15.8	67.0 ± 15.5	57.5 ± 17.8	61.1 ± 18.2	65.7 ± 8.6	0.10
Male sex (*n*, %)	70, (64.8%)	19, (70.4%)	17, (63.0%)	15, (55.6)	19, (70.4%)	0.62
Time on PD (months)	25.9 ± 28.5	19.3 ± 15.9	28.3 ± 37.1	29.6 ± 35.7	26.2 ± 19.1	0.56
Mode of PD (*n*, %)						0.16
Continuous ambulatory PD (*n*, %)	52, (48.1%)	18, (66.7%)	12, (44.4%)	11, (40.7%)	11, (40.7%)	
Automated PD (*n*, %)	56, (51.9%)	9, (33.3%)	15, (55.6%)	16, (59.3%)	16, (59.3%)	
BMI (kg/m^2^)	26.4 ± 4.5	26.2 ± 3.6	25.9 ± 4.2	26.0 ± 4.9	27.6 ± 5.0	0.47
Presence of diabetes (*n*, %)	39, (36.1%)	11, (40.7%)	7, (25.9%)	8, (29.6%)	13, (48.1%)	0.30
Pre-existing cardiovascular disease (*n*, %)	48, (44.4%)	18, (66.7%)	12, (44.4%)	6, (22.2%)	12, (44.4%)	<0.05
Hemoglobin (g/dL)	11.5 ± 1.5	11.6 ± 1.8	11.9 ± 1.1	11.7 ± 1.6	10.9 ± 1.2	0.08
Serum albumin (g/dL)	3.7 ± 0.4	3.6 ± 0.5	3.8 ± 0.3	3.8 ± 0.4	3.6 ± 0.4	0.07
Antihypertensive drug use (*n*, %)	98, (90.7%)	24 (88.9%)	24 (88.9%)	25, (92.6%)	25, (92.6%)	0.55
ACEIs or ARBs (*n*, %)	43, (39.8%)	3, (11.1%)	12, (44.4%)	12, (44.4%)	16, (59.3%)	0.001
CCBs (*n*, %)	61, (56.5%)	6, (22.2%)	16, (59.3%)	18, (66.7%)	21, (77.8%)	<0.001
β-blockers (*n*, %)	90, (83.3%)	22, (81.5%)	23, (85.2%)	21, (77.8%)	24, (88.9%)	0.72
Clinic SBP (mmHg)	132.9 ± 19.4	112.3 ± 11.4	130.6 ± 12.7	135.6 ± 13.5	153.0 ± 14.3	<0.001
Clinic DBP (mmHg)	78.0 ± 12.9	69.7 ± 9.9	74.8 ± 12.8	83.6 ± 11.6	84.0 ± 11.8	<0.001

Abbreviations: ACEi = angiotensin-converting-enzyme-inhibitor; ARB = angiotensin-receptor-blocker; BMI = body mass index; CCB = calcium-channel-blocker; DBP = diastolic blood pressure; PD = peritoneal dialysis.

**Table 2:** Hazard ratio for the composite outcome of non-fatal MI, non-fatal stroke, or all-cause death according to the quartile of clinic and 24-h ambulatory SBP.

SBP		Unadjusted Analysis			Adjusted Analysis *		
Clinic	Range (mmHg)	HR	95% CI	*p* Value	HR	95% CI	*p* Value
Quartile 1	<119.2	1			1		
Quartile 2	119.2–132.0	0.201	0.057–0.711	<0.05	0.255	0.069–0.940	<0.05
Quartile 3	132.0–145.7	1.028	0.486–2.176	0.94	1.472	0.651–3.331	0.35
Quartile 4	>145.7	1.750	0.851–3.598	0.13	1.648	0.766–3.547	0.20
		Model fit (χ^2^): 16.5 *p* = 0.001			Model fit (χ^2^): 42.0 *p* < 0.001		
**24-h Ambulatory**							
Quartile 1	<114.0	1			1		
Quartile 2	114.0–126.0	0.667	0.280–1.586	0.36	1.098	0.434–2.777	0.84
Quartile 3	126.0–140.7	0.558	0.228–1.367	0.20	1.004	0.382–2.635	0.99
Quartile 4	>140.7	2.240	1.103–4.547	<0.05	2.449	1.156–5.190	<0.05
		Model fit (χ^2^): 18.4 *p* < 0.001			Model fit (χ^2^): 40.3 *p* < 0.001		

Abbreviations: CI = confidence interval; HR = hazard ratio; SBP = systolic blood pressure; * Adjusted analysis for both clinic and 24-h ambulatory SBP models included the following variables: age, sex, dialysis vintage, PD modality, diabetic status, pre-existing cardiovascular disease (defined as prior MI, prior stroke or history of congestive heart failure), presence of substantial residual diuresis, antihypertensive drug use, serum albumin, and hemoglobin levels.

**Table 3:** Hazard ratio for the composite outcome of non-fatal MI, non-fatal stroke, or all-cause death according to the quartile of daytime and nighttime ambulatory SBP.

Ambulatory SBP		Unadjusted Analysis			Adjusted Analysis *		
Daytime	Range (mmHg)	HR	95% CI	*p* Value	HR	95% CI	*p* Value
Quartile 1	<114.2	1			1		
Quartile 2	114.2–126.5	0.503	0.198–1.280	0.15	0.854	0.313–2.328	0.76
Quartile 3	126.5–142.7	0.651	0.281–1.508	0.32	1.061	0.427–2.637	0.89
Quartile 4	>142.7	2.646	1.292–5.422	<0.01	2.631	1.247–5.535	0.01
		Model fit (χ^2^): 25.0 *p* < 0.001			Model fit (χ^2^): 43.9 *p* < 0.001		
**Nighttime**							
Quartile 1	<108.2	1			1		
Quartile 2	108.2–123.0	0.357	0.135–0.939	<0.05	0.594	0.214–1.650	0.32
Quartile 3	123.0–140.0	0.619	0.271–1.413	0.26	1.555	0.575–4.206	0.38
Quartile 4	>140.0	1.886	0.947–3.759	0.07	2.305	1.047–5.072	<0.05
		Model fit (χ^2^): 19.5 *p* < 0.001			Model fit (χ^2^): 40.6 *p* < 0.001		

Abbreviations: CI = confidence interval; HR = hazard ratio; SBP= systolic blood pressure. * Adjusted analysis for both daytime and nighttime ambulatory SBP models included the following variables: age, sex, dialysis vintage, PD modality, diabetic status, pre-existing cardiovascular disease (defined as prior MI, prior stroke or history of congestive heart failure), presence of substantial residual diuresis, antihypertensive drug use, serum albumin, and hemoglobin levels.

## Data Availability

Data available upon request due to restrictions e.g., privacy or ethical.

## Supplementary Materials

The following are available online at https://www.mdpi.com/article/10.3390/jcm10112232/s1, Table S1: Distribution of study participants across quartiles of standardized and 24-h ambulatory SBP.
