# Supplementary material for: The Relation of Clinic and Ambulatory BP with the Risk of Cardiovascular Events and All-Cause Mortality among Patients on Peritoneal Dialysis
Source: J Clin Med. 2021 May 21;10(11):2232. doi: 10.3390/jcm10112232 (PMC8196741; doi:10.3390/jcm10112232)
Supplement: Supplementary file 1 [file jcm-10-02232-s001.zip › jcm-1174950-supplementary.pdf]

Supplementary Materials

Table 1. Distribution of study participants across quartiles of standardized and 24-hour ambulatory SBP.

Quartiles of clinic SBP (mmHg)	Quartiles of 24-hour ambulatory SBP (mmHg)				Total
	Q4 (>140.7)	Q3 (126.0–140.7)	Q2 (114.0–126.0)	Q1 (<114.0)	
Q4 (>145.7)	19	4	4	0	27
Q3 (132.0–145.7)	6	13	7	1	27
Q2 (119.2–132.0)	2	8	9	8	27
Q4 (<119.2)	0	2	7	18	27
Total	27	27	27	27	108
